# Supplementary material for: Pseudorabies virus inhibits progesterone-induced inactivation of TRPML1 to facilitate viral entry
Source: PLoS Pathog. 2024 Jan 31;20(1):e1011956. doi: 10.1371/journal.ppat.1011956 (PMC10829982; doi:10.1371/journal.ppat.1011956)
Supplement: S1 Table — (DOCX) [file ppat.1011956.s002.docx]

**Table S1. List of primers used in this study.**

| **Genes** | **Forward (**5′-3′**)** | **Reverse (**5′-3′**)** |
| --- | --- | --- |
| PR | ATGTTGTATAAGG | CTTTTTATGAAAGAGAAG |
| TRPML1 | ATGGCAGTCCCCGTAGG | ATTCACCAGCAGCGA |
| TPCN1 | ATGGCTGTGAGTTTA | GGTGATGGTCTGGGA |
| TPCN2 | ATGGCGGAGCCCCAG | CCTGCACAGCTGCA |
| MDM2 | ATGTGCAATACCAAC | GGGGAAATAAGTTAG |
| TRIM21 | ATGGCCTCAGCACTGC | CCAGCCCGTCCTCAGTG |
| RC3H2 | GCTTCCACAATGCCTGTGCAG | ACCTCCTTGCTGTTAACCAT |
| Mouse Q-PTGS2 | CATCCCCTTCCTGCGAAGTT | GGCCCTGGTGTAGTAGGAGA |
| Mouse Q-SCT | AGCGAGCAGGACACAGAAAA | AGAGAGACAGGGACCCATCC |
| Mouse Q-β-actin | CCCCATTGAACATGGCATTG | ACGACCAGAGGCATACAGG |
| PRV Q-gH | CTCGCCATCGTCAGCAA | GCTGCTCCTCCATGTCCTT |
| Mouse Q-GNRHR | GGCATCAGGCCTTCTACAACT | GCTGTAGTTTGCGTGGGTCT |
| Mouse Q-LHCGR | TAGCCACTGCTGTGCTTTCA | TCGTAATCCCAGCCACTGAG |
| Mouse Q-FSHR | GTGCATTCAACGGAACCCAG | AGGGAGCTTTTTCAAGCGGT |
| Mouse Q-TRPML1 | CATGTTTGTGACGTTCGCGG | AGTACCTCCTGGGTGCTTGA |
| Pig Q-TRPML1 | CACCTTTGACATCGACCCGA | TGACGTTGATCAGCTTGTGGA |
| Pig Q-IFN-β | CTCTAGCACTGGCTGGAATGAA | CCGGAGGTAATCTGTAAGTCTGT |
| Pig Q-IL-6 | GCCTGAGGGCCATTCGGATA | TGTGCCCAGTGGACAGGTTT |
| Pig Q-TPCN1 | CTTAGGAAGGAGGAACAGGCG | CCACTACCTCTCCCCTGGTAA |
| Pig Q-TPCN2 | ATGGCAGGAGACTTTGCTCC | AATCTCGGCTTTTCGAGCCA |
| Pig Q- PIKfyve | CCCAGGAGAACACAGACACC | GTCACTGTCCTGGCTCTTCC |
| Pig Q-MTM1 | CCCGGACTGGGAGAGTTTC | TCCCGATTAACTCCGTCCCT |
| Pig Q-Fig4 | GTTCAAGGTGCCCAGATGGA | CTTTCCCAAGGGGCTGCATA |
| Pig Q-β-actin | CTGAACCCCAAAGCCAACCGT | TTCTCCTTGATGTCCCGCACG |
