# Supplementary material for: Pseudorabies virus inhibits progesterone-induced inactivation of TRPML1 to facilitate viral entry
Source: PLoS Pathog. 2024 Jan 31;20(1):e1011956. doi: 10.1371/journal.ppat.1011956 (PMC10829982; doi:10.1371/journal.ppat.1011956)
Supplement: S2 Table — (DOCX) [file ppat.1011956.s003.docx]

**Table S2. List of sgRNAs and shRNAs used in this study.**

| **Genes** | **Forward (**5′-3′**)** | **Reverse (**5′-3′**)** |
| --- | --- | --- |
| sg-PGR-1 | GCGCTGGGACGCCCAGACAC | GTGTCTGGGCGTCCCAGCGC |
| sg-PGR-2 | GGAAGCGTCGCCCGCAGCCT | AGGCTGCGGGCGACGCTTCC |
| sh-TRPML1-1 | GCTACCCAACAGATTCCTACG | CGTAGGAATCTGTTGGGTAGC |
| sh-TRPML1-2 | GCTGGATGCACTGGTCTTAGA | TCTAAGACCAGTGCATCCAGC |
| sh-MDM2-1 | GCACAGAAAGACACTTATACT | AGTATAAGTGTCTTTCTGTGC |
| sh-MDM2-2 | GCGCCACAAATCTGATAATAT | ATATTATCAGATTTGTGGCGC |
