# Supplementary figures and images for: Pseudorabies virus inhibits progesterone-induced inactivation of TRPML1 to facilitate viral entry
Source: PLoS Pathog. 2024 Jan 31;20(1):e1011956. doi: 10.1371/journal.ppat.1011956 (PMC10829982; doi:10.1371/journal.ppat.1011956)

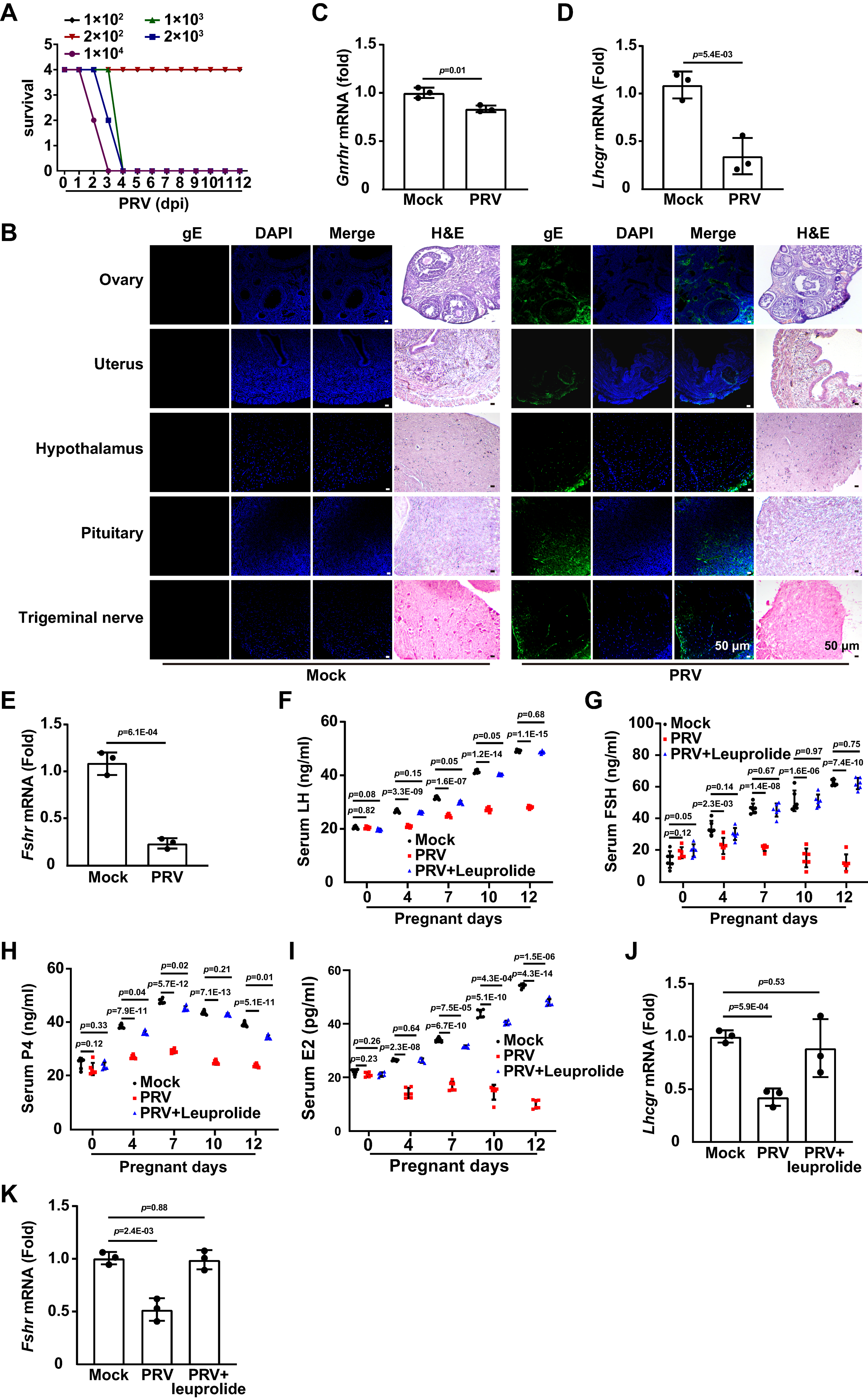

Supplement: S1 Fig — (A) Female C57BL/6J mice were intranasally infected with PRV HN1201 (1 × 10^2^–1 × 10^4^ TCID50 per mouse). Survival rate was monitored daily for 12 days (n = 12). (B) Female C57BL/6J mice were intranasally infected with PRV HN1201 (2 × 10^2^ per mouse) for 2 days. PRV gE expression in the ovary, uterus, hypothalamus, pituitary and trigeminal nerve was detected by immunohistochemistry. PRV-induced mouse histopathology was performed by hematoxylin and eosin staining. Scale bar: 50 μm. (C–E) The mRNA levels of GnRHR in the pituitary (C), and LHCGR (D) and FSHR (E) in the ovary of mock-infected or PRV-infected female mice were analyzed by qRT-PCR analysis at 2 days post pregnancy (n = 3). (F–I) LH (F), FSH (G) P4 (H) and E2 (I) in the serum of indicated female mice were quantified by ELISA assay at indicated pregnant days (n = 6). (J and K) The mRNA levels of LHCGR (J) and FSHR (K) in the ovary of indicated female mice were analyzed by qRT-PCR analysis at 4 days post pregnancy (n = 3). Data are expressed as the mean ± SD of 3 independent experiments. P-values were determined by Student’s t-test. P < 0.05 was considered statistically significant. (TIF) [file ppat.1011956.s004.tif]

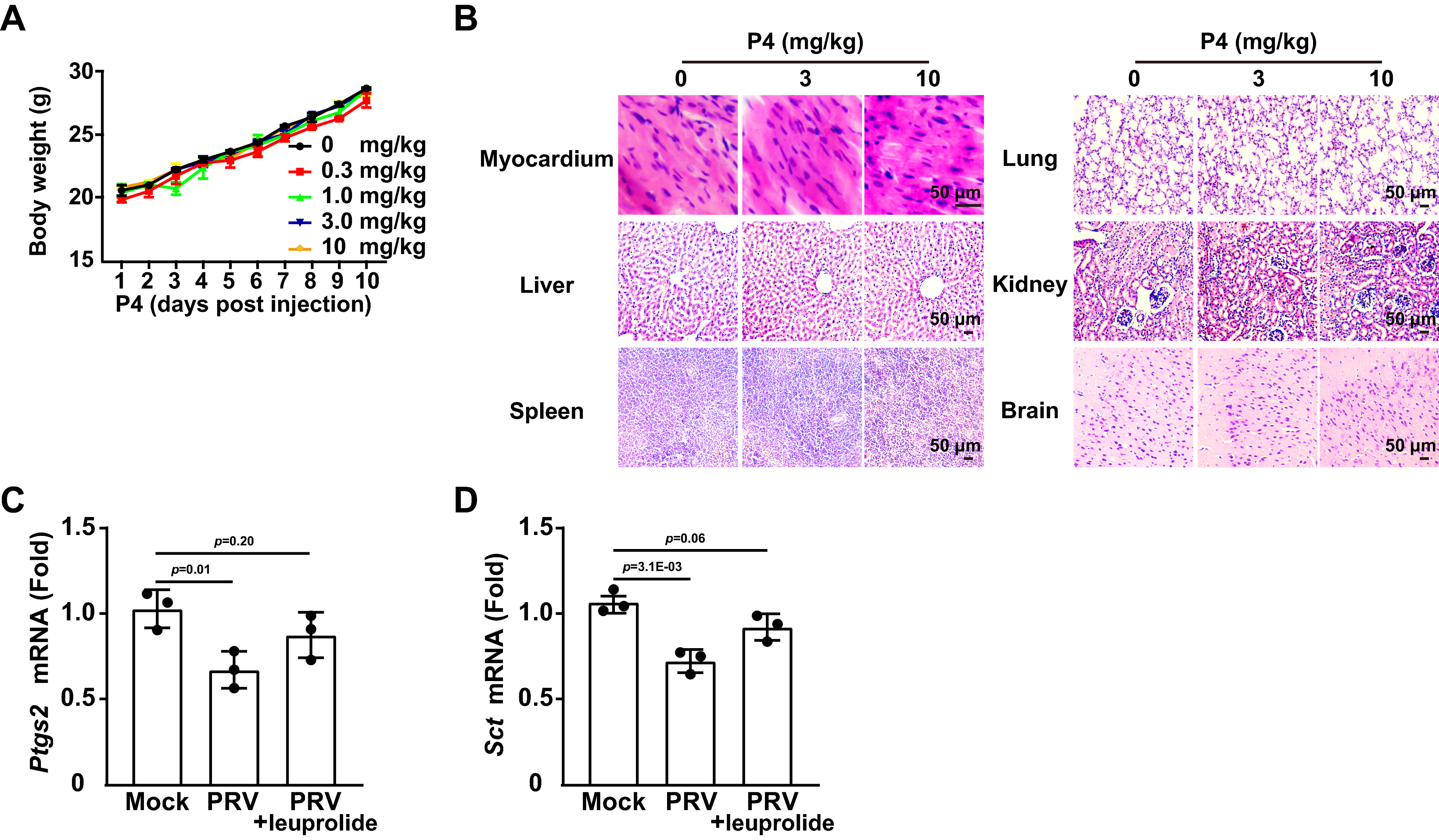

Supplement: S2 Fig — (A) Female C57BL/6J mice were injected daily with the indicated concentrations of P4. Body weight was measured from days 1 to 10 (n = 10). (B) F Female C57BL/6J mice were injected daily with P4 at doses of 0, 3, and 10 mg/kg per mouse. Hematoxylin and eosin staining was performed on sections from the myocardium, liver, spleen, lung, kidney, and brain at 10 days post-injection. Scale bar: 50 μm. (C and D) On day -1, female C57BL/6J mice were either mock-infected or intranasally infected with PRV HN1201 (2 × 10^2^ TCID50 per mouse). On day 0, the mock-infected or PRV-infected female mice were mated with male mice overnight. On day 2, the female mice were injected with leuprolide (1.5 mg/kg). On day 4, the mRNA levels of PTGS2 (C) and SCT (D) in the ovary were analyzed by qRT-PCR analysis. Data are expressed as the mean ± SD of 3 independent experiments. P-values were determined by Student’s t-test. P < 0.05 was considered statistically significant. (TIF) [file ppat.1011956.s005.tif]

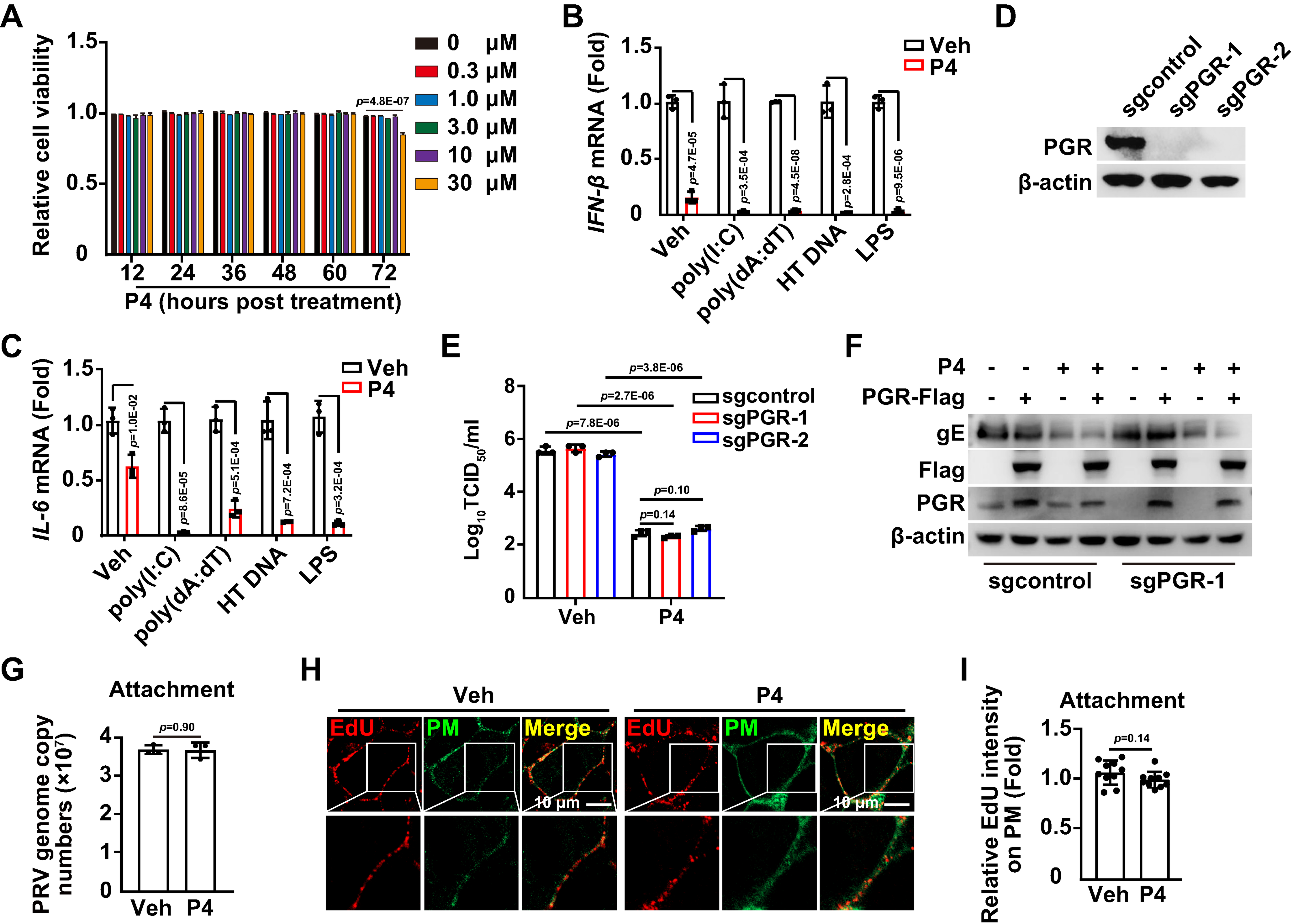

Supplement: S3 Fig — (A) PK-15 cells were treated with the indicated concentrations of P4 (0–30 μM) for 12–72 hours. Cell viability was assessed by a CCK-8 assay. (B and C) PK-15 cells treated with vehicle, poly(I:C) (5 μg/mL), poly(dA:dT) (5 μg/mL), HT-DNA (2 μg/mL), or LPS (10 μg/mL) for 24 hours. The mRNA levels of IFN-β (B) and IL-6 (C) were analyzed by qRT-PCR. (D) Immunoblotting was performed to detect PGR in sgcontrol and sgPGR PK-15 cells. (E) sgcontrol and sgPGR PK-15 cells were infected with PRV HN1201 (MOI = 0.1) and treated with P4 (10 μM) for 24 hours. Viral titer was determined by a TCID50 assay. (F) sgcontrol and sgPGR PK-15 cells were transfected with the PGR-Flag plasmid for 24 hours. The cells were then infected with PRV HN1201 (MOI = 0.1) and treated with P4 (10 μM) as indicated for 24 hours. PRV gE, PGR-Flag, and PGR were analyzed by immunoblotting. (G) PK-15 cells were incubated with PRV HN1201 (MOI = 0.1) and P4 (10 μM) at 4°C for 2 hours. Viral attachment was detected by qRT-PCR analysis of viral genome copy numbers on the PM. (H) PK-15 cells were incubated with EdU-labeled PRV HN1201 (MOI = 0.1) and P4 (10 μM) at 4°C for 2 hours. Viral attachment was observed using Apollo staining (red). DiI (green, 20 μM) indicated the PM. Scale bar: 10 μm. (I) Quantification of the relative EdU intensity on the PM of PK-15 cells from (H). Data are expressed as the mean ± SD of 3 independent experiments. P-values were determined by Student’s t-test. P < 0.05 was considered statistically significant. (TIF) [file ppat.1011956.s006.tif]

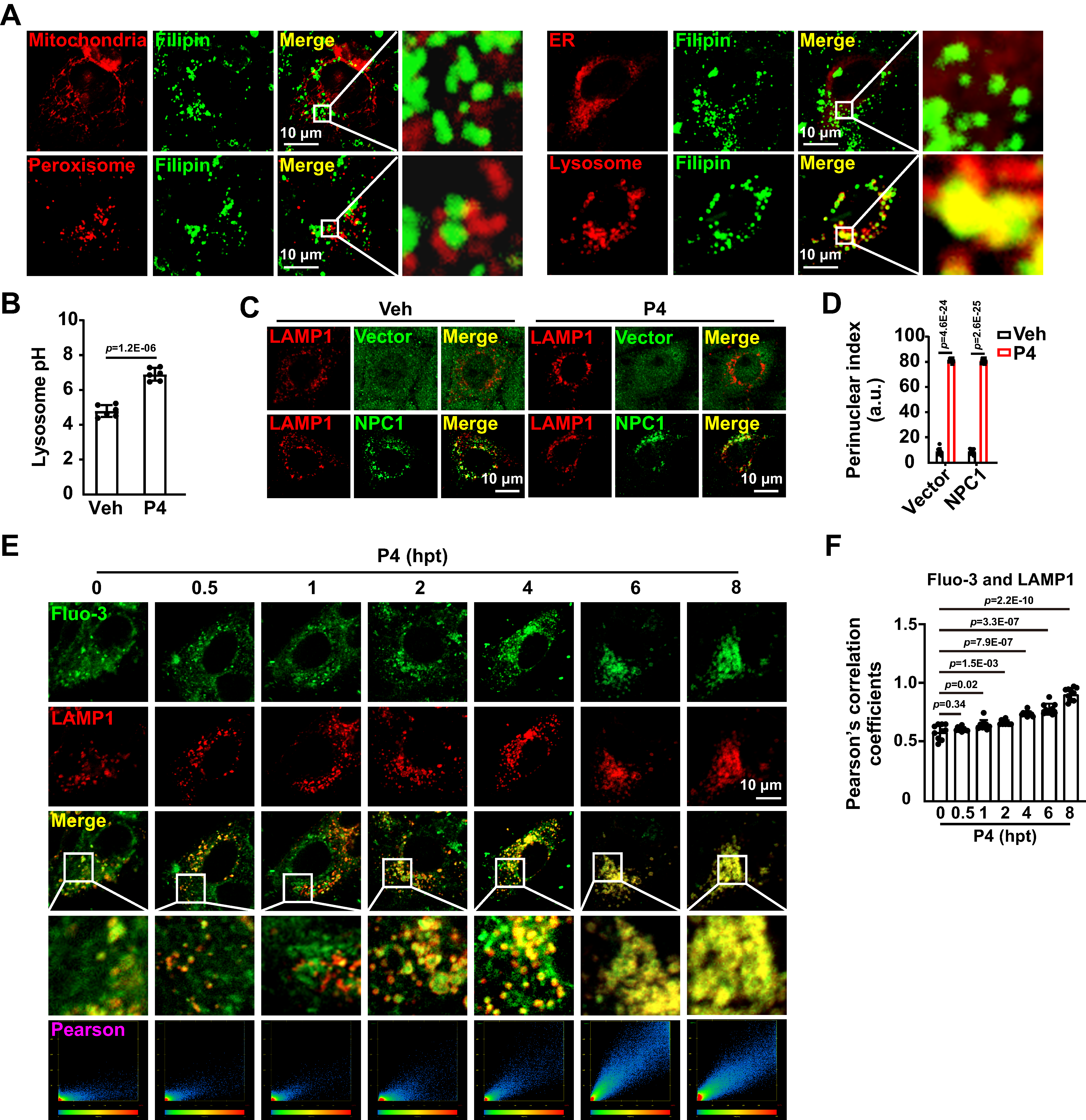

Supplement: S4 Fig — (A) PK-15 cells were treated with P4 (10 μM) for 8 hours. Co-localization analysis of intracellular cholesterol (filipin) with mitochondria, peroxisomes, endoplasmic reticulum (ER), and lysosomes was performed by immunofluorescence and filipin staining. Scale bar: 10 μm. (B) PK-15 cells were treated with vehicle or P4 (10 μM). Lysosomal pH was measured 8 hours post-treatment. (C) PK-15 cells were transfected with a vector or NPC1-GFP plasmid for 24 hours. Afterward, cells were treated with vehicle or P4 (10 μM) for an additional 8 hours. Co-localization analysis of LAMP1 (red) with NPC1-GFP was performed by immunofluorescence analysis. Scale bar: 10 μm. (D) Quantification of the perinuclear index of LAMP1 in PK-15 cells from (C). (E) PK-15 cells were treated with P4 (10 μM) for 0–8 hours. Co-localization analysis of fluo-3 with LAMP1 was performed by immunofluorescence analysis. Scale bar: 10 μm. (F) Pearson’s correlation coefficients of fluo-3 with LAMP1 in PK-15 cells from (E). Data are expressed as the mean ± SD of 3 independent experiments. P-values were determined by Student’s t-test. P < 0.05 was considered statistically significant. (TIF) [file ppat.1011956.s007.tif]

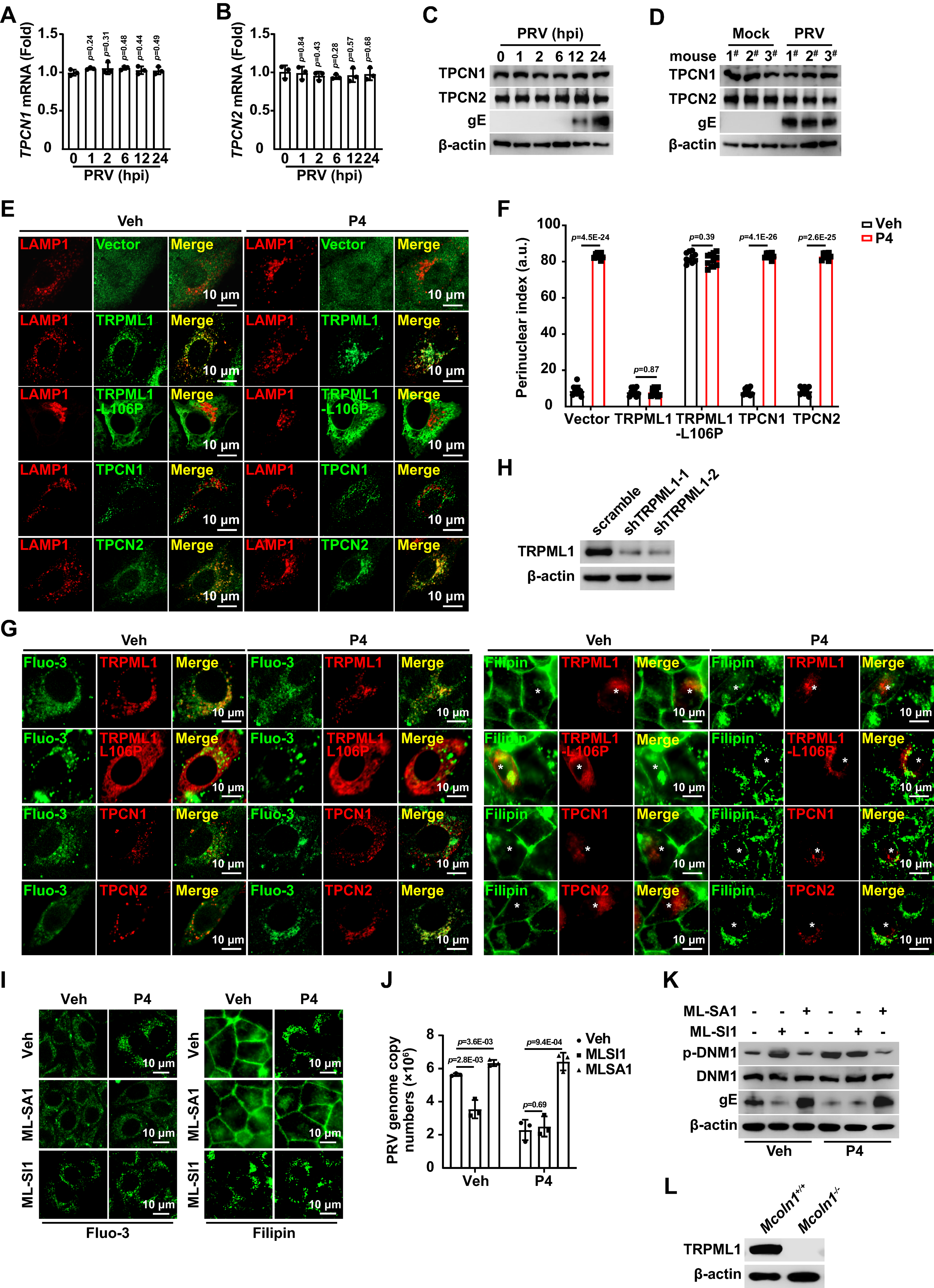

Supplement: S5 Fig — (A and B) PK-15 cells were infected with PRV HN1201 (MOI = 0.1) for 0–24 hours. The mRNA levels of TPCN1 (A) and TPCN2 (B) were analyzed by qRT-PCR. (C) PK-15 cells were treated as indicated in (A). TPCN1 and TPCN2 proteins were analyzed by immunoblotting. (D) Mice were either mock-infected or intranasally infected with PRV (2 × 10^3^ TCID50 per mouse) for 2 days. TPCN1 and TPCN2 in the uterus were analyzed by immunoblotting (n = 3). (E) PK-15 cells were transfected with TRPML1-FLAG, TRPML1-FLAG L106P, TPCN1-FLAG, and TPCN2-FLAG plasmids and treated with vehicle or P4 (10 μM) for 24 hours. Colocalization of LAMP1 with TRPML1 variants, TPCN1, and TPCN2 was analyzed by immunofluorescence analysis. Scale bar: 10 μm. (F) Quantification of the perinuclear index of LAMP1 in PK-15 cells from (E). (G) PK-15 cells were transfected with TRPML1-mCherry, TRPML1-mCherry L106P, TPCN1-mCherry, and TPCN2-mCherry and treated with vehicle or P4 (10 μM) for 24 hours. Colocalization of fluo-3 and intracellular cholesterol (filipin) with TRPML1 variants, TPCN1, and TPCN2 was analyzed by immunofluorescence. Scale bar: 10 μm. (H) TRPML1 in scramble, shTRPML1-1, and shTRPML1-2 was detected by immunoblotting. (I) PK-15 cells were treated with P4 (10 μM), ML-SA1 (20 μM), and ML-SI1 (10 μM) as indicated. Fluo-3 and filipin staining were performed 8 hours post-treatment. Scale bar: 10 μm. (J) PK-15 cells were incubated with PRV HN1201 (MOI = 0.1) and treated with vehicle, P4 (10 μM), ML-SA1 (20 μM), or ML-SI1 (10 μM) as indicated at 4°C for 2 hours. Cells were then shifted to 37°C for 10 minutes to allow entry. After washing with trypsin (1 mg/mL) to remove residual virions on the PM, viral entry was detected by qRT-PCR analysis of viral genome copy numbers in the cells. (K) PK-15 cells were treated with vehicle, P4 (10 μM), ML-SA1 (20 μM), or ML-SI1 (10 μM) as indicated for 8 hours and then infected with PRV HN1201 (MOI = 0.1) for 24 hours. p-DNM1, DNM1, and PRV gE were detected by immunoblotti [file ppat.1011956.s008.tif]

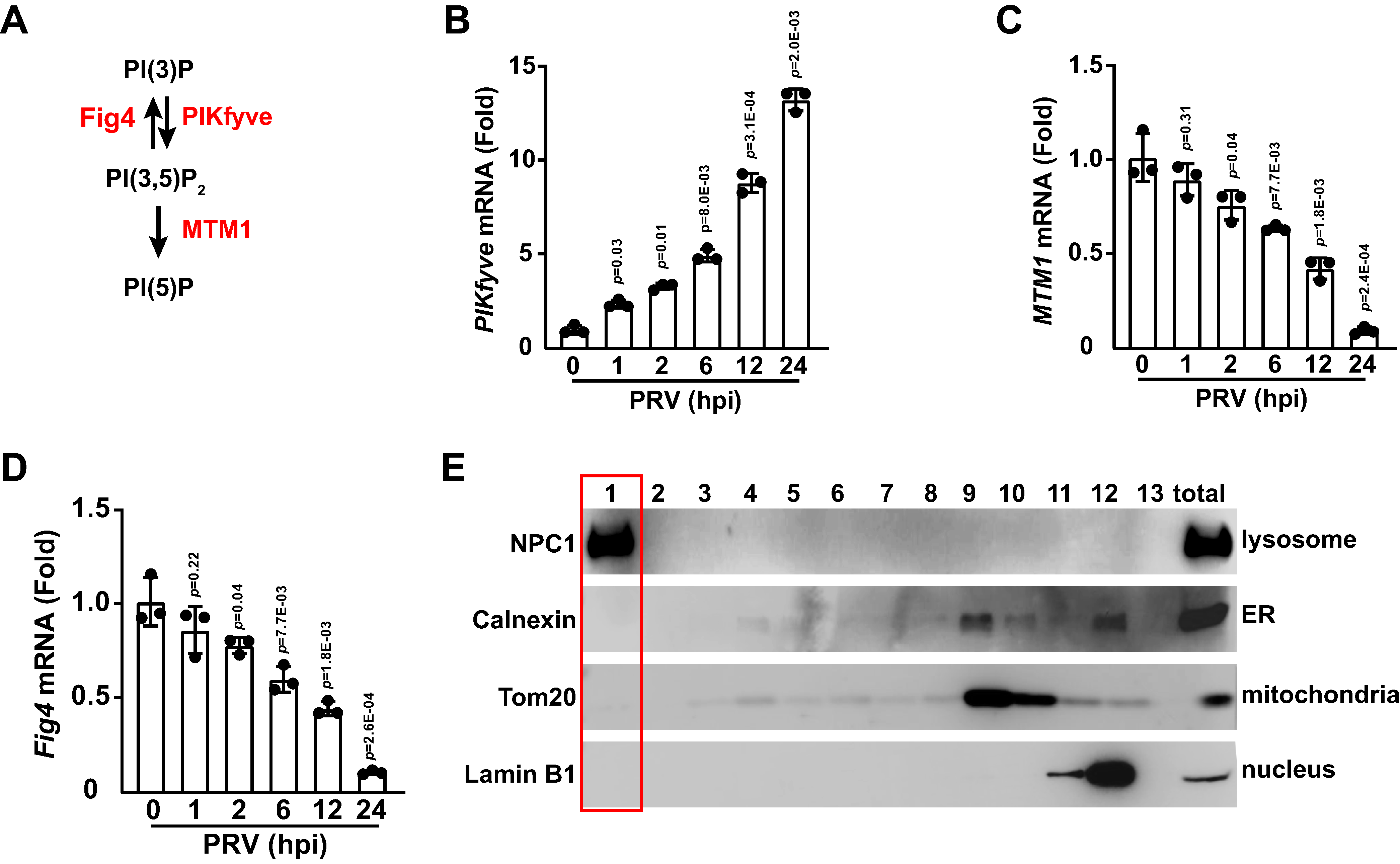

Supplement: S6 Fig — (A) Schematic diagram of the PI(3,5)P2 metabolic pathways. (B–D) PK-15 cells were infected with PRV HN1201 (MOI = 0.1) for the indicated times. The mRNA levels of PIKfyve (B), MTM1 (C), and Fig 4 (D) were analyzed by qRT-PCR analysis. (E) Immunoblotting analysis of NPC1 (lysosome), Calnexin (ER), Tom20 (mitochondria), and Lamin B1 (nucleus) in PK-15 cell lysates subjected to iodixanol density gradient centrifugation. Data are expressed as the mean ± SD of 3 independent experiments. P-values were determined by Student’s t-test. P < 0.05 was considered statistically significant. (TIF) [file ppat.1011956.s009.tif]

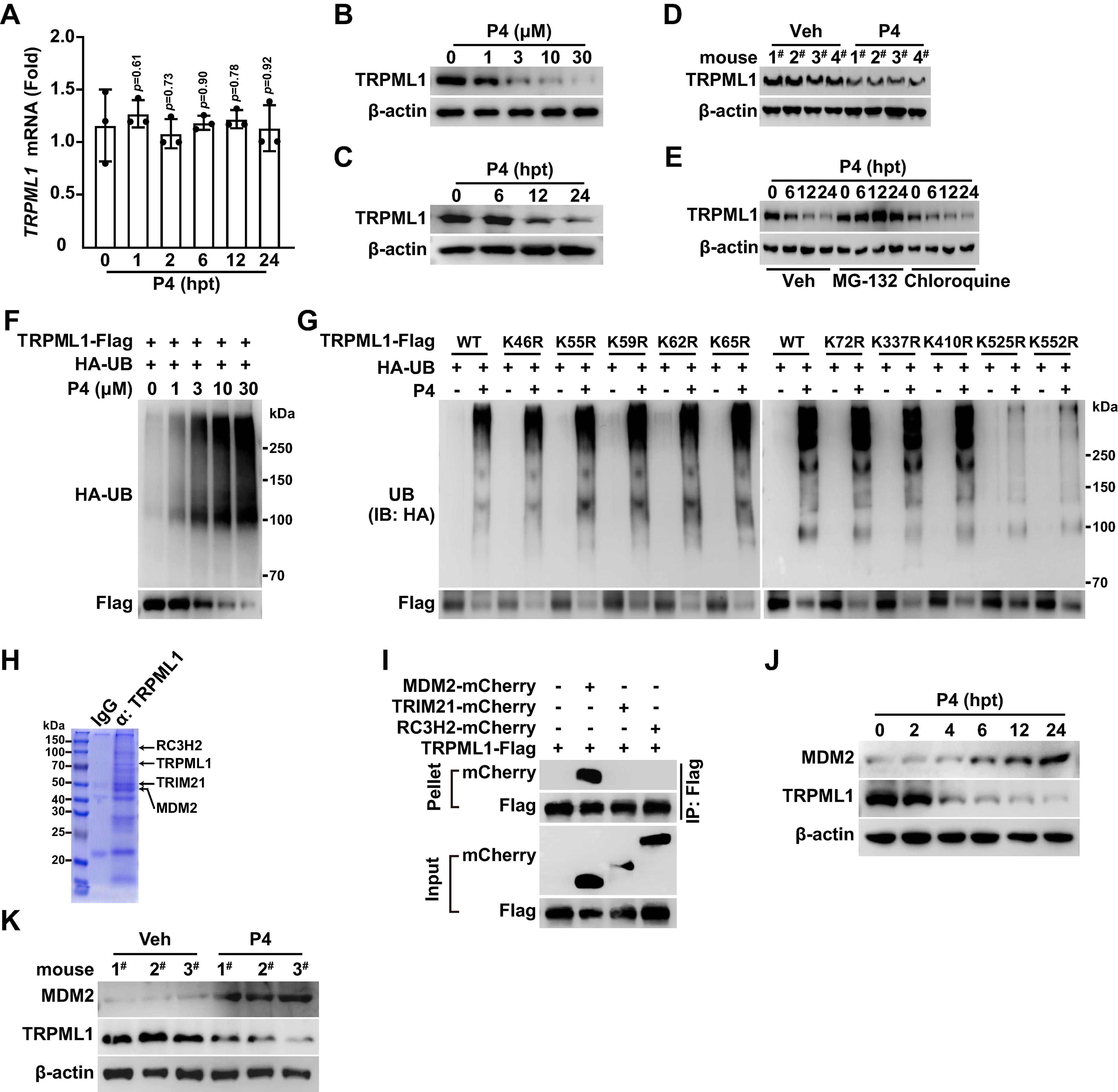

Supplement: S7 Fig — (A) PK-15 cells were treated with P4 (10 μM) for 0–24 hours. The mRNA levels of TRPML1 were analyzed by qRT-PCR. (B) PK-15 cells were treated with P4 (0–30 μM) for 24 hours. TRPML1 levels were analyzed by immunoblotting. (C) PK-15 cells were treated with P4 (10 μM) for 0–24 hours. TRPML1 levels were analyzed by immunoblotting. (D) Mice were injected daily with vehicle or P4 (10 mg/kg per mouse) for 10 days. TRPML1 levels in the uterus were analyzed by immunoblotting (n = 3). (E) PK-15 cells were treated with vehicle, P4 (10 μM), MG-132 (10 μM), or chloroquine (10 μM) as indicated for 0–24 hours. TRPML1 levels were analyzed by immunoblotting. (F) PK-15 cells were transfected with TRPML1-FLAG and HA-UB plasmids, and treated with P4 (0–30 μM) as indicated for 24 hours. Ubiquitination of TRPML1-FLAG was analyzed by ubiquitination assay. (G) PK-15 cells were transfected with TRPML1-FLAG variants and HA-UB plasmids, and treated with P4 (0–30 μM) for 24 hours. Ubiquitination of TRPML1-FLAG was analyzed by ubiquitination assay. (H) Coomassie blue staining was used to detect potential E3 ubiquitin ligases associated with TRPML1. (I) PK-15 cells were transfected with TRPML1-FLAG and the indicated E3 plasmids for 24 hours. The interactions of TRPML1-FLAG with the indicated E3 ligases were analyzed by CoIP analysis. (J) PK-15 cells were treated with P4 (10 μM) for the indicated times. MDM2 and TRPML1 levels were analyzed by immunoblotting. (K) MDM2 and TRPML1 levels in the uterus from experiment D were analyzed by immunoblotting. Data are expressed as the mean ± SD of 3 independent experiments. P-values were determined by Student’s t-test. P < 0.05 was considered statistically significant. (TIF) [file ppat.1011956.s010.tif]
