# Supplementary material for: Pseudorabies virus inhibits progesterone-induced inactivation of TRPML1 to facilitate viral entry
Source: PLoS Pathog. 2024 Jan 31;20(1):e1011956. doi: 10.1371/journal.ppat.1011956 (PMC10829982; doi:10.1371/journal.ppat.1011956)
Supplement: Attachment — Submitted filename: Response to reviewers.docx [file ppat.1011956.s011.docx]

**Responses to reviewers**

**Part I – Summary**

**Reviewer #1**

*In this manuscript, the authors used pseudorabies virus (PRV), a swine alphaherpesvirus, and mice model to address why PRV infection causes reproductive failure (infertility). Their data showed that PRV infection affected the hypothalamus-pituitary-ovary axis (HPOA) and induced infertility by downregulating progesterone P4, and further revealed the molecular pathway contributing this phenomenon, proposing a novel mechanism by which PRV hijacks the lysosomal pathway to evade P4-mediated antiviral defense and impair female fertility. This study is of general significance for explaining the impact of viral diseases on female reproductive health and for developing antiviral therapies to control the associated reproduction issues clinically caused by virus infection. The work is well designed and complete, and the data supporting the conclusion are basically sufficient.*

**Response:** We appreciate the positive comments from the reviewer on our manuscript.

**Reviewer #2**

*In the submitted manuscript, Su et al. investigates the important regulatory role of P4 in the process of infertility induced by PRV virus. Importantly, endo-lysosomal channel TRPML1 as a host factor to facilitate viral entry was demonstrated, and P4 can induce proteasomal degradation of TRPML1 via MDM2 to inhibit viral entry, which reveals a novel mechanism of how PRV influenced P4 to induce infertility and provides a new strategy for developing antiviral therapies. Overall, the study contributes a new knowledge on the role of P4 and TRPML1 in viral endosomal entry pathways. The conclusions are backed up by a lot of data. However, there are some points that would need addressing before publication.*

**Response:** We appreciate the positive comments from the reviewer on our manuscript. Some points are addressed in part II and part III.

**Reviewer #3**

**Strengths**

*1. Identification that PRV facilitates lysosomal-dependent viral entry via TRPML1*

*2. Exploration of the effects of leuprolide in this infection model is interesting*

**Response:** We thank for these two strengths pointed out by the reviewer.

**Weaknesses**

*1. Insufficient details regarding the mouse model of PRV infection*

**Response:** We thank for the reviewer’s suggestion. The details regarding the mouse model of PRV infection were described in the figure legends in the revised manuscript as follow:

**Figure 1A:** Schematic diagram of experimental procedure for PRV-induced infertility *in vivo*. On day -1, female C57BL/6J mice were mock infected or intranasally infected with PRV HN1201 (2 × 10^2^ TCID_50_ per mouse). On day 0, mock infected or PRV-infected female mice were mated with male mice overnight. On day 2, the mRNA levels of GnRHR in the pituitary, and LHCGR and FSHR in the ovary were analyzed by qRT-PCR analysis. On day 4–12, GnRH, LH, FSH, P4 and E2 levels in serum were quantified by ELISA. The analysis of pregnant rate and embryo numbers were determined on day 12. Liter size was counted on day 23.

**Figure 1J:** Schematic diagram of experimental procedure for assessing the effect of leuprolide on PRV-induced infertility *in vivo*. On day -1, female C57BL/6J mice were mock infected or intranasally infected with PRV HN1201 (2 × 10^2^ TCID_50_ per mouse). On day 0, mock infected or PRV-infected female mice were mated with male mice overnight. On day 2, female mice were injected with leuprolide (1.5 mg/kg). On day 4, the mRNA levels of GnRHR in the pituitary, and LHCGR and FSHR in the ovary were analyzed by qRT-PCR analysis. On day 4–12, GnRH, LH, FSH, P4 and E2 levels in serum were quantified by ELISA. Female mice were additionally injected with leuprolide (1.5 mg/kg) on day 9. The analysis of pregnant rate and embryo numbers were determined on day 12. Liter size was counted on day 23.

**Figure 2A:** Schematic diagram of experimental procedure for assessing the effect of P4 on PRV-induced infertility *in vivo*. On day -1, female C57BL/6J mice were mock infected or intranasally infected with PRV HN1201 (2 × 10^2^ TCID_50_ per mouse). On day 0, mock infected or PRV-infected female mice were mated with male mice overnight. On day 2, female mice were injected with P4 (10 mg/kg per mouse) every day for 10 days. On day 4–12, GnRH, LH, FSH, P4 and E2 levels in serum were quantified by ELISA. The analysis of pregnant rate and embryo numbers were determined on day 12. Liter size was counted on day 23.

*2. Missed opportunity to explore the effects of PRV infection in mouse CNS and reproductive organs*

**Response:** We thank for the reviewer’s suggestion. To better delineate the histopathology induced by PRV infection in mice, we performed immunohistochemical staining for PRV gE and hematoxylin and eosin staining of mouse tissues. PRV gE expression was detected in tissue sections from the ovary, uterus, hypothalamus, pituitary, and trigeminal nerve at two days post-infection with PRV (**Figure S1B**). Noticeable histopathological changes were observed in the ovary, uterus, pituitary, and trigeminal nerve, but not in the hypothalamus (**Figure S1B**).

**Figure S1B**

*3. Without better delineation of PRV-induced mouse histopathology, initial studies are largely observational and the more mechanistic studies (i.e., effects of PRV infection on TRPML1 expression and lysosomal synthesis of PI synthesis) lack sufficient context to determine relevancy*

**Response:** We thank for the reviewer’s suggestion. To better delineate the histopathology induced by PRV infection in mice, we performed immunohistochemical staining for PRV gE and hematoxylin and eosin staining of mouse tissues. PRV gE expression was detected in tissue sections from the ovary, uterus, hypothalamus, pituitary, and trigeminal nerve at two days post-infection with PRV (**Figure S1B**). Noticeable histopathological changes were observed in the ovary, uterus, pituitary, and trigeminal nerve, but not in the hypothalamus (**Figure S1B**).

**Figure S1B**

*4. Insufficient explanation for* *the rising* *FSH levels measured in mock-infected controls during the first 12 days of pregnancy*

**Response:** Djati MS and colleagues have measured FSH levels in serum of healthy pregnant mice [1]. They detect FSH levels are 24.55±1.73 ng/ml (day 8), 51.30±0.67 (day 12) and 29.12±2.74 (day 16), suggesting FSH levels are steadily rising during the first 12 days of pregnancy. In our present study, the pattern of rising FSH levels measured in mock-infected controls during the first 12 days of pregnancy were consistent with the above findings. We added the explanation in the revised manuscript.

*5. While manuscript contains few grammatical errors it is sometimes difficult to follow and more importantly lacks adequate explanation of the significance of its findings*

**Response:** We thank for the reviewer’s suggestion. Grammatical errors were corrected and adequate explanation of the significance of our findings were added in the revised manuscript.

*6. Figure panel size makes data interpretation challenging and legends do not provide sufficient detail (including but not limited to statistical considerations)*

**Response:** We thank for the reviewer’s suggestion. We used more than one assay to interpreted one phenomenon. Therefore, this makes large panel size. We improved the data interpretation in the revised manuscript. Sufficient details of legends were also provided in the revised manuscript.

**Part II – Major Issues: Key Experiments Required for Acceptance**

**Reviewer #1: No**

**Reviewer #2**

*1. In Fig. 1, it was shown that* *2 of 12 were pregnant in the PRV-infected group, why the Embryo number of PRV-infected mice was 3? Did the authors put the embryos of different batches of pregnant and virus-infected mice together?*

**Response:** We did not put the embryos of different batches of pregnant and virus-infected mice together. 12 female mice were mated with male mice in the mock infected and PRV-infected group (**Figure 1B**). We found that 2 of 12 female mice were pregnant in the PRV-infected group, suggesting PRV infection affected pregnancy.

Furthermore, we counted the embryo number from the PRV-infected pregnant mice and found that PRV infection reduce embryo number and litter size (**Figure 1C and 1D**). These data indicated that PRV infection affected not only pregnancy, but also embryo development.

*2. In Fig. 5, the authors have shown that PRV infection upregulated TRPML1 expression and the TRPML1 protein plays an important role in viral entry. Previous studies have found that endosomal cation channel* *TRPML2 (mBio. 2018 Jan 30; 9(1): e02314-17. doi: 10.1128/mBio.02314-17), as a key host factor, can also promote viral entry by enhancing the efficiency of viral trafficking. Why did the authors not* *consider the response of other TRPML proteins to PSV?*

**Response:** TRPML1, TRPML2, and TRPML3 are the three members of the TRPML subfamily of ion channels. Each of these channels is encoded by a distinct gene: MCOLN1 codes for TRPML1, MCOLN2 codes for TRPML2, and MCOLN3 codes for TRPML3 [2].

TRPML1 is the best-characterized TRPML1member. It is broadly expressed across various tissues, indicating that TRPML1 has general functions essential for many cell types. TRPML1 is directly implicated in mucolipidosis type IV, a neurodegenerative lysosomal storage disorder, which results from loss-of-function mutations in the MCOLN1 gene [3]. We observed that P4 induced cholesterol accumulated in the lysosome (**Figure 4A**), which is a phenotype of lysosomal lipid storage diseases belonged to lysosomal storage disorder [4]. This data suggested that TRPML1 might be involved in P4-induced lysosomal lipid storage diseases.

TRPML2 expression is more restricted and less understood. It is found in certain immune cell populations, such as monocytes and dendritic cells, suggesting a role in the immune system [5]. Previous studies as the reviewer suggested (mBio. 2018 Jan 30; 9(1): e02314-17. doi: 10.1128/mBio.02314-17) indicate that TRPML2 can promote viral entry by enhancing the efficiency of viral trafficking. They used human monocyte THP-1 cells as a model, whereas we used porcine kidney epithelial PK-15 cells in our recent study.

TRPML3 is expressed in a variety of tissues but is most notably present in hair cells of the inner ear, as well as in skin cells where it's linked to pigmentation. The channel is implicated in sensory processes such as hearing and balance, as mutations in the corresponding gene can cause hearing loss and vestibular disorders in mice and potentially humans [6].

Considering of the widespread expression pattern and the involvement in mucolipidosis type IV of TRPML1, we suggested that TRPML1 might function relevant with P4-induced lysosomal cholesterol accumulation. Therefore, we did not consider the response of other TRPML proteins to PRV.

*3. In Fig. 4 and Fig. 5, it was shown that PRV infection stimulated significant Ca2+ increases, and this enhancement was mainly through TRPML1 channel regulated by PRV infection. The effect of TRPML1 channel activity on viral infection should be investigated.*

**Response:** L106P mutations of TRPML1 have been identified in human Mucolipidosis IV patients, suggesting this mutant is absent for TRPML1 channel activity [7]. We generated *Mcoln1^-/-^* MEF cells from *Mcoln1^-/-^* mice embryo and performed rescue experiment by introducing wild type TRPML1 and TRPML1 L106P in the cells. Comparable levels of viral entry and replication were detected in TRPML1-WT-expressing *Mcoln1^+/+^* and *Mcoln1^-/-^* MEF cells (**Figure 5I and 5J, vehicle group**). Viral entry and replication were inhibited in *Mcoln1^+/+^* and *Mcoln1^-/-^* MEF cells transfected with TRPML1-L106P (**Figure 5I and 5J, vehicle group**). These data indicated that the effect of TRPML1 channel activity was essential for PRV infection.

**Figure 5I and 5J**

In addition, TRPML1 activation requires its endogenous activator PI(3,5)P_2_ [8]. In **Figure 6**, we demonstrate that PRV infection promotes lysosomal PI(3,5)P_2_ synthesis to promote lysosomal Ca^2+^ efflux. We also used TRPML1-7Q mutant with seven substitutions (R42Q/R43Q/R44Q/K55Q/R57Q/R61Q/K62Q), which is unable to bind to PI(3,5)P_2_, to confirm the activation of TRPML1 through PI(3,5)P_2_ by Ca^2+^ efflux (**Figure 6N**). These results can also confirm the effect of TRPML1 channel activity was essential for PRV infection.

**Figure 6N**

*4. PRV infection not only reduces the level of P4, but also promotes the expression of TRPML1 and intracellular calcium transport. How to distinguish and verify which process happens first on a time scale? At the same time, in addition to affecting TRPML1 expression, whether P4 can also affect TRPML1-mediated intracellular calcium and cholesterol accumulation?*

**Response:** The present study demonstrated that infection of mice with PRV affected HPOA-regulated P4 levels, which prevented MDM2-mediated proteasomal degradation of TRPML that can be stimulated by PRV infection. Enhanced activation of TRPML1 subsequently promoted lysosomal cholesterol and Ca^2+^ efflux to facilitate PRV entry (**Figure S8**).

**Figure S8**

In **Figure S5G**, we showed that TRPML1-L106P was unable to release the accumulated Ca^2+^ and cholesterol from the lysosomes, while TRPML1 WT did. This result suggested that in addition to affecting TRPML1 expression, P4 can also affect TRPML1-mediated intracellular calcium and cholesterol accumulation.

**Figure S5G**

**Reviewer #3**

*Figure 1 provides underpinning for studies - that PRV infection disrupts HPO axis function. As displayed in panel G, mock-infected controls show serum levels of FSH that rise in linear fashion from day 0 - day 12 of pregnancy. This result to me is unexpected. It would be expected for FSH levels to be highest on day 0 and at parturition (not measured in this study) and also that rising concentrations of estrogen and progesterone during pregnancy would inhibit FSH release and lower FSH levels from their day 0 peak. Manuscript does not address FSH results in the mock-infected controls or possible reasons why mice in their study demonstrate steadily rising levels of FSH during pregnancy.*

**Response:** Djati MS and colleagues have measured FSH levels in serum of healthy pregnant mice [1]. They detect FSH levels are 24.55±1.73 ng/ml (day 8), 51.30±0.67 (day 12) and 29.12±2.74 (day 16), suggesting FSH levels are steadily rising during the first 12 days of pregnancy. In our present study, the pattern of rising FSH levels measured in mock-infected controls during the first 12 days of pregnancy were consistent with the above findings. We added the explanation in the revised manuscript. We measured the level of estradiol, using ELISA assay in the serum of mock- or PRV-infected mice. The ELISA results showed that level of serum estradiol was steadily increased in mock-infected mice during the 12-day pregnancy (**Figure 1I**).

**Figure 1I**

*In mice, PRV causes CNS symptoms and high mortality. After oronasal inoculation, PRV replicates in nasal epithelium and enters free nerve endings in TG and other neurons innervating nasal mucosa. PRV replication in the cell bodies of TG is followed by axonal transport to second-order neurons in the afferent trigeminal nuclei of the brain stem. Experimental detail regarding lethality should be included (especially as mice were infected with PRV HN1201, a strain that induces more severe infection in swine compared to the classical Fa strain).*

**Response:** We thank for the reviewer’s suggestion. Experimental detail regarding lethality have been included in **Figure S1A**. Female C57BL/6J mice were intranasally infected with PRV HN1201 (ranging from 1 × 10^2^–1 × 10^4^ TCID_50_ per mouse) for 12 days. No mice died when they were challenged with PRV at doses of 1 × 10^2^ and 2 × 10^2^ TCID_50_ (**Figure S1A**). Therefore, in subsequent experiments, we intranasally infected the mice with PRV HN1201 at a dose of 2 × 10^2^ TCID_50_.

**Figure S1A**

*Likewise, it will be important to assess histopathology of PRV infection in CNS tissue (including the hypothalamus and pituitary) and reproductive tissue (PRV causes viremia that can seed endometrial tissue and ovaries). These studies are need to clarify the effects of PRV infection on the hypothalamus or pituitary vs. ovary vs. PRV-induced fetal demise (all of which have the potential to reduce circulating levels of progesterone.*

**Response:** We thank for the reviewer’s suggestion. To better delineate the histopathology induced by PRV infection in mice, we performed immunohistochemical staining for PRV gE and hematoxylin and eosin staining of mouse tissues. PRV gE expression was detected in tissue sections from the ovary, uterus, hypothalamus, pituitary, and trigeminal nerve at two days post-infection with PRV (**Figure S1B**). Noticeable histopathological changes were observed in the ovary, uterus, pituitary, and trigeminal nerve, but not in the hypothalamus (**Figure S1B**).

**Figure S1B**

**Part III – Minor Issues: Editorial and Data Presentation Modifications**

**Reviewer #1**

*1. Only a alphaherpesvirus (PRV) was used in this study. The title should define “Pseudorabies virus”.*

**Response:** The title has been changed to “Pseudorabies virus inhibits progesterone-induced inactivation of TRPML1 to facilitate viral entry”.

*2. In the results,* *a summary for the data and a diagram describing the mechanism should be provided as the last paragraph of this section in order to help readers understand the findings.*

**Response:** We thank for the reviewer’s suggestion. a summary for the data and a diagram describing the mechanism has been provided in the results.

*3. There are some inappropriate descriptions and grammar uses in the manuscript.*

**Response:** The inappropriate descriptions and grammar uses have been corrected.

**Reviewer #2**

*1. In mouse model, why did the author* *set 12 days as the detection time of mouse pregnancy?*

**Response:** Pregnancy can be clearly seen from the mice at 12 days post-mating. Mouse embryonic fibroblast cells were usually prepared by using 12-15 days’ embryo [9]. In addition, we showed that the defects of embryo development induced by PRV infection could be observed at 12 days’ pregnancy (Figure 1C and 1L). Therefore, we set 12 days as the detection time of mouse pregnancy.

*2. In Fig. 1 and 2, the author has introduced the downstream of hypothalamus-pituitary-ovary axis includes estrogen and P4, why only P4 level was tested and verified, and did PSV infection cause changes in estrogen level?*

**Response:** We thank for the reviewer’s suggestion. We measured the level of estradiol (E2), which showed that E2 was l increased in mock-infected mice during the 12-day pregnancy (**Figures 1I**). In contrast, PRV infection resulted in significant decreases in the levels of GnRH, LH, FSH, P4, and E2 (**Figures 1I**).

**Figures 1I**

*3. In Fig. 5C, why is there no detectable protein from the virus in the first six hours?*

**Response:** The PRV gE protein is involved in various aspects of the virus lifecycle and has several functions: viral replication and packaging, cell-to-cell spread, viral egress and antigenicity [10]. The expression of gE is regulated during the infection cycle and is typically produced in the later stages of infection. Therefore, there is no detectable gE protein from the virus in the first six hours.

*4. Abbreviations should be introduced upon first mention.*

**Response:** We thank for the reviewer’s suggestion. Abbreviations have been introduced upon first mention.

*5. In Fig. 1, why does P4 level decrease less significantly to* *GnRH, LH, FSH in hypothalamus-pituitary-ovary axis?*

**Response:** In our present research, we showed that PRV-induced infertility was mainly caused by a decrease in P4 level, but not by decreases in GnRH, LH, FSH and E2 levels (**Figure 2**). Given our data indicated that the liter size reduced to approximately 50% in PRV-infected group compared to control group (**Figure 1D**), we suggested that the significant less decreased P4 level to GnRH, LH, FSH was closely related with PRV-induced infertility. If P4 level decreased more significantly to GnRH, LH, FSH, severe reduced liter size could be observed by PRV infection. In addition, except for the generation of P4 through HPOA, placenta and adrenal glands are also the source of progesterone production, which may be a reason why P4 level decreases less significantly to GnRH, LH, FSH.

*6. The schematic model showing PRV evades P4-mediated antiviral defense and impairs female fertility in Fig. S8 should be moved to the main manuscript.*

**Response:** We thank for the reviewer’s suggestion. The schematic model showing PRV evades P4-mediated antiviral defense and impairs female fertility in Fig. S8 has been moved to the main manuscript.

*7. P12 line 250 - rephrase this sentence - " However, MCOLN1 was transcriptionally activated by PRV infection (Figure 5A and 5B). TRPML1 protein level was also increased in PRV-infected cells or in a murine uterus".*

**Response:** We rephrased this sentence as follow: However, MCOLN1, which encodes the TRPML1 protein, was transcriptionally activated by PRV infection (Figures 5A and 5B). TRPML1 protein levels also increased in PRV-infected cells, as well as in murine uterus (Figures 5C and 5D).

*8. The authors have shown that PRV infection specifically resulted in translocation of lysosomal cholesterol and Ca2+ to the PM, how does increased calcium concentration promote the viral entry.*

**Response:** Ca^2+^ released from lysosomes can activate dynamin via calcineurin, facilitating the fission and separation of endocytic vesicles from the plasma membrane, thereby promoting endocytosis [11]. Dynamin (DNM) mediates vesicle fission from the plasma membrane during endocytosis, while its dephosphorylation activates the intrinsic GTPase activity, which depends on Ca^2+^ [27]. For this reason, we analyzed DNM1 phosphorylation by immunoblotting during viral entry. DNM1 became dephosphorylated as early as 5 min post-viral entry (**Figure 4L**). No change in DNM1 phosphorylation was observed in PRV-infected cells treated with P4 for 0–20 min (**Figure 4L**). These results indicate that P4 inhibits DNM dephosphorylation to prevent viral entry.

*9. I do think a professional copy edit is still required, and it would make the manuscript much smoother to read.*

**Response:** We thank for the reviewer’s suggestion. A professional copy edit is performed.

**Reviewer #3**

*Data is poorly displayed throughout manuscript and legend content lacks sufficient detail throughout. These are major rather than minor concerns as it makes manuscript difficult to evaluate. As an additional major concern, sentence construction is sometime disjointed and various statements in the introduction and discussion lack cogency and precision. As example, lines 381-384:*

*"**Pregnant women are the largest group of adults with disseminated HSV infection. The overall HSV seroprevalence among pregnant women is 72%. The mean gestational age at presentation is 31 weeks and the case fatality rate is 39% for both mothers and neonates."*

*These 3 sentences seem to have little to do with each other and with the research presented in the manuscript. If they are included in the discussion, tt would also be useful to provide reference for the statements that pregnant women are the largest group of adults with disseminated HSV infection and that rates of fatality are identical among HSV-infected women and neonates.*

**Response:** We thank for the reviewer’s suggestion. As the reviewer mentioned that “These 3 sentences seem to have little to do with each other and with the research presented in the manuscript.”, we deleted these 3 sentences in discussion. The revised manuscript has been improved according to the reviewer’s suggestion. Sufficient detail of legend content has been added.

**References:**

1.	Djati MS, Christina YI, Rifa'i M. The combination of Elephantopus scaber and Sauropus androgynus promotes erythroid lineages and modulates follicle-stimulating hormone and luteinizing hormone levels in pregnant mice infected with Escherichia coli. Vet World. 2021;14(5):1398-404. Epub 20210531. doi: 10.14202/vetworld.2021.1398-1404. PubMed PMID: 34220146; PubMed Central PMCID: PMCPMC8243661.

2.	Himmel NJ, Cox DN. Transient receptor potential channels: current perspectives on evolution, structure, function and nomenclature. Proc Biol Sci. 2020;287(1933):20201309. Epub 20200826. doi: 10.1098/rspb.2020.1309. PubMed PMID: 32842926; PubMed Central PMCID: PMCPMC7482286.

3.	Wang W, Zhang X, Gao Q, Xu H. TRPML1: an ion channel in the lysosome. Handb Exp Pharmacol. 2014;222:631-45. doi: 10.1007/978-3-642-54215-2_24. PubMed PMID: 24756723.

4.	Schulze H, Sandhoff K. Lysosomal lipid storage diseases. Cold Spring Harb Perspect Biol. 2011;3(6). Epub 20110601. doi: 10.1101/cshperspect.a004804. PubMed PMID: 21502308; PubMed Central PMCID: PMCPMC3098676.

5.	Cuajungco MP, Silva J, Habibi A, Valadez JA. The mucolipin-2 (TRPML2) ion channel: a tissue-specific protein crucial to normal cell function. Pflugers Arch. 2016;468(2):177-92. Epub 20150904. doi: 10.1007/s00424-015-1732-2. PubMed PMID: 26336837; PubMed Central PMCID: PMCPMC4715775.

6.	Grimm C, Barthmes M, Wahl-Schott C. Trpml3. Handb Exp Pharmacol. 2014;222:659-74. doi: 10.1007/978-3-642-54215-2_26. PubMed PMID: 24756725.

7.	Sun M, Goldin E, Stahl S, Falardeau JL, Kennedy JC, Acierno JS, Jr., et al. Mucolipidosis type IV is caused by mutations in a gene encoding a novel transient receptor potential channel. Hum Mol Genet. 2000;9(17):2471-8. doi: 10.1093/hmg/9.17.2471. PubMed PMID: 11030752.

8.	Dong XP, Shen D, Wang X, Dawson T, Li X, Zhang Q, et al. PI(3,5)P(2) controls membrane trafficking by direct activation of mucolipin Ca(2+) release channels in the endolysosome. Nat Commun. 2010;1(4):38. Epub 20100713. doi: 10.1038/ncomms1037. PubMed PMID: 20802798; PubMed Central PMCID: PMCPMC2928581.

9.	Tan YS, Lei YL. Generation and Culture of Mouse Embryonic Fibroblasts. Methods Mol Biol. 2019;1960:85-91. doi: 10.1007/978-1-4939-9167-9_7. PubMed PMID: 30798523; PubMed Central PMCID: PMCPMC6533614.

10.	Pomeranz LE, Reynolds AE, Hengartner CJ. Molecular biology of pseudorabies virus: impact on neurovirology and veterinary medicine. Microbiol Mol Biol Rev. 2005;69(3):462-500. doi: 10.1128/MMBR.69.3.462-500.2005. PubMed PMID: 16148307; PubMed Central PMCID: PMCPMC1197806.

11.	Liu JP, Sim AT, Robinson PJ. Calcineurin inhibition of dynamin I GTPase activity coupled to nerve terminal depolarization. Science. 1994;265(5174):970-3. doi: 10.1126/science.8052858. PubMed PMID: 8052858.
